# Postoperative Visual Acuity Outcomes After Phacoemulsification in Patients With Type 2 Diabetes Mellitus: A Systematic Review

**DOI:** 10.7759/cureus.99901

**Published:** 2025-12-23

**Authors:** Osman Haji, Ling Paulina Gronczewska, Ahmed Alahmad

**Affiliations:** 1 General Medicine, National Health Service (NHS) England, London, GBR; 2 General Medicine, West Suffolk Hospital, Bury St Edmunds, GBR; 3 Department of Gastroenterology, Luton and Dunstable Hospital, Luton, GBR

**Keywords:** cataract, diabetes mellitus type 2, phacoemulsification, postoperative, visual acuity

## Abstract

Cataract surgery is commonly performed in people with type 2 diabetes mellitus (T2DM), but concerns persist regarding postoperative visual recovery even before the onset of diabetic retinopathy. Understanding outcomes in this population is essential for evidence-based counselling and perioperative management. Our objective in this systematic review was to evaluate postoperative visual acuity outcomes following phacoemulsification in adults with T2DM without diabetic retinopathy or diabetic macular oedema, compared with non-diabetic individuals. A systematic search of Web of Science, Scopus, and PubMed was conducted in May 2025, from database inception. Eligible studies included English-language prospective comparative studies reporting postoperative visual acuity outcomes following phacoemulsification in T2DM patients without pre-existing proliferative diabetic retinopathy or macular disease, compared with non-diabetic controls. Study selection, data extraction, and risk-of-bias assessment using the Newcastle-Ottawa Scale were performed independently and in duplicate. Of 121 records screened, 11 full texts were assessed, and two studies met the inclusion criteria. The two included prospective comparative cohort studies involved 137 eyes in patients with T2DM and 137 eyes in non-diabetic controls. Both studies demonstrated substantial postoperative improvement in best-corrected visual acuity in both groups. Subclinical postoperative corneal and macular changes were more frequent in diabetics in one study, without a meaningful impact on functional vision over the reported follow-up. Risk of bias was moderate in both studies due to residual confounding and short-term follow-up. In conclusion, patients with T2DM without retinopathy experience excellent post-operative visual outcomes after phacoemulsification, comparable to those of non-diabetic individuals. Vigilant perioperative monitoring remains warranted due to increased susceptibility to transient corneal and retinal changes. High-quality studies with longer follow-up and appropriate adjustment for diabetic severity are required to refine prognostic accuracy and confirm long-term outcome equivalence.

## Introduction and background

Cataract remains the leading cause of reversible visual impairment worldwide [1] and poses a particularly significant clinical burden among individuals with type 2 diabetes mellitus (T2DM) [2]. The global prevalence of diabetes continues to rise, and a proportional increase in cataract surgery demand is expected as these patients develop lens opacities earlier and more aggressively than the non-diabetic population [3]. Hyperglycaemia-induced biochemical changes, including advanced glycation end-product accumulation, polyol pathway activation, oxidative stress, and osmotic dysregulation within lens fibres, collectively accelerate cataract formation in diabetes [4] and can negatively influence postoperative recovery [5].

Phacoemulsification with posterior chamber intraocular lens implantation is widely regarded as the standard of care for visually significant cataract due to its reproducibility, minimal tissue disruption, and rapid visual rehabilitation [6]. However, diabetic eyes possess several inherent vulnerabilities that may affect surgical outcomes, such as compromised corneal endothelial function, altered tear film stability, delayed wound healing responses, and retinal microvascular compromise [7].

The impact of established diabetic retinopathy and diabetic macular oedema on postoperative visual outcomes is well documented [8,9]. Presence of either condition is associated with a higher risk of suboptimal acuity, postoperative macular oedema, and prolonged recovery [10]. Consequently, careful retinal evaluation is routine in diabetic patients undergoing preoperative assessment [11]. A substantial proportion of patients, however, present for cataract surgery without any clinical evidence of retinopathy [12]. In such cases, the primary question facing clinicians concerns whether diabetes in isolation confers an elevated risk for poorer postoperative vision.

A focused synthesis of studies comparing phacoemulsification outcomes specifically in T2DM patients without diabetic retinopathy or DME against non-diabetic controls is therefore essential. Such a review addresses a common clinical scenario and can inform decision-making regarding patient counselling, perioperative risk stratification, and postoperative monitoring protocols. Furthermore, by critically appraising the methodological quality of the available evidence, the review can identify current knowledge gaps and guide future research priorities. The objective of this systematic review is to evaluate postoperative visual acuity outcomes following phacoemulsification in adults with T2DM without evidence of diabetic retinopathy or macular disease, compared with non-diabetic individuals, and to interpret these findings in the context of clinical relevance and service-wide implications.

## Review

Methods

Protocol and Reporting

This systematic review was conducted in accordance with the Preferred Reporting Items for Systematic Reviews and Meta-Analyses (PRISMA) guidelines. The review question was structured around a predefined eligibility framework based on the Population, Intervention, Comparator, and Outcomes (PICO). A review protocol was developed a priori.

Eligibility Criteria

The population, intervention, comparator, and outcome are shown in Table 1.

**Table 1:** PICO framework PICO: Population, Intervention, Comparator, and Outcomes, OCT: Optical coherence tomography.

PICO	Criteria
Population	Adults with type 2 diabetes mellitus undergoing phacoemulsification for age-related cataract. Only studies that explicitly excluded diabetic retinopathy of any stage, as well as diabetic macular edema or any other macular pathology, were eligible.	
Intervention	Phacoemulsification with posterior chamber intraocular lens implantation.	
Comparator	Non-diabetic individuals undergoing the same procedure.	
Outcomes	Postoperative visual acuity measured as best-corrected visual acuity (BCVA), uncorrected visual acuity where applicable, and reported in Snellen or logMAR notation. Secondary anatomical outcomes (e.g. OCT macular thickness, endothelial cell metrics) were noted where relevant to functional outcomes.	

Study Design and Other Criteria

Eligible designs included prospective comparative cohort studies and case-control studies. Studies were restricted to English, human subjects, and full-text availability. Retrospective papers, case reports, case series without a comparator, editorials, reviews, and studies involving combined ocular procedures were excluded. Studies involving type 1 diabetics were also excluded.

Information Sources and Search Strategy

A comprehensive electronic search was conducted in Web of Science, Scopus, and PubMed from database inception to May 2025. Search terms combined Medical Subject Headings (MeSH) and free text related to diabetes, phacoemulsification, cataract surgery, and visual outcomes. Reference lists of included studies were manually screened to identify any additional eligible reports.

Study Selection

Search results were imported into screening software and duplicate records were removed. Titles and abstracts were screened independently and in duplicate by two reviewers, followed by full-text review of potentially eligible articles. Conflicts were resolved through discussion and consensus, with a third reviewer weighing in if necessary.

Data Extraction

Two reviewers independently extracted data into a predefined structured table. Extracted variables included study design, location, sample sizes, patient demographics, phacoemulsification technique, postoperative follow-up schedule, visual acuity outcomes, and secondary anatomical outcomes. Where logMAR values were not reported, Snellen values were to be converted using standard formulas when necessary.

Risk-of-Bias Assessment

Risk of bias for non-randomised studies was assessed independently by two reviewers using the Newcastle-Ottawa Scale (NOS), evaluating domains of selection, comparability, and outcome assessment. Disagreements were resolved by consensus. NOS results were summarised narratively and presented in tabular form.

Data Synthesis

A narrative synthesis approach was employed because of the limited number of eligible studies, methodological heterogeneity, and variation in postoperative follow-up intervals and visual acuity reporting formats, which would make meta-analysis difficult. Findings were synthesised according to outcome type and timepoint, with particular emphasis on the clinical meaningfulness of between-group differences. This included assessing postoperative visual acuity, as well as secondary physiological effects such as impact on the retina and cornea. 

Results

Study Selection

**Figure 1:**
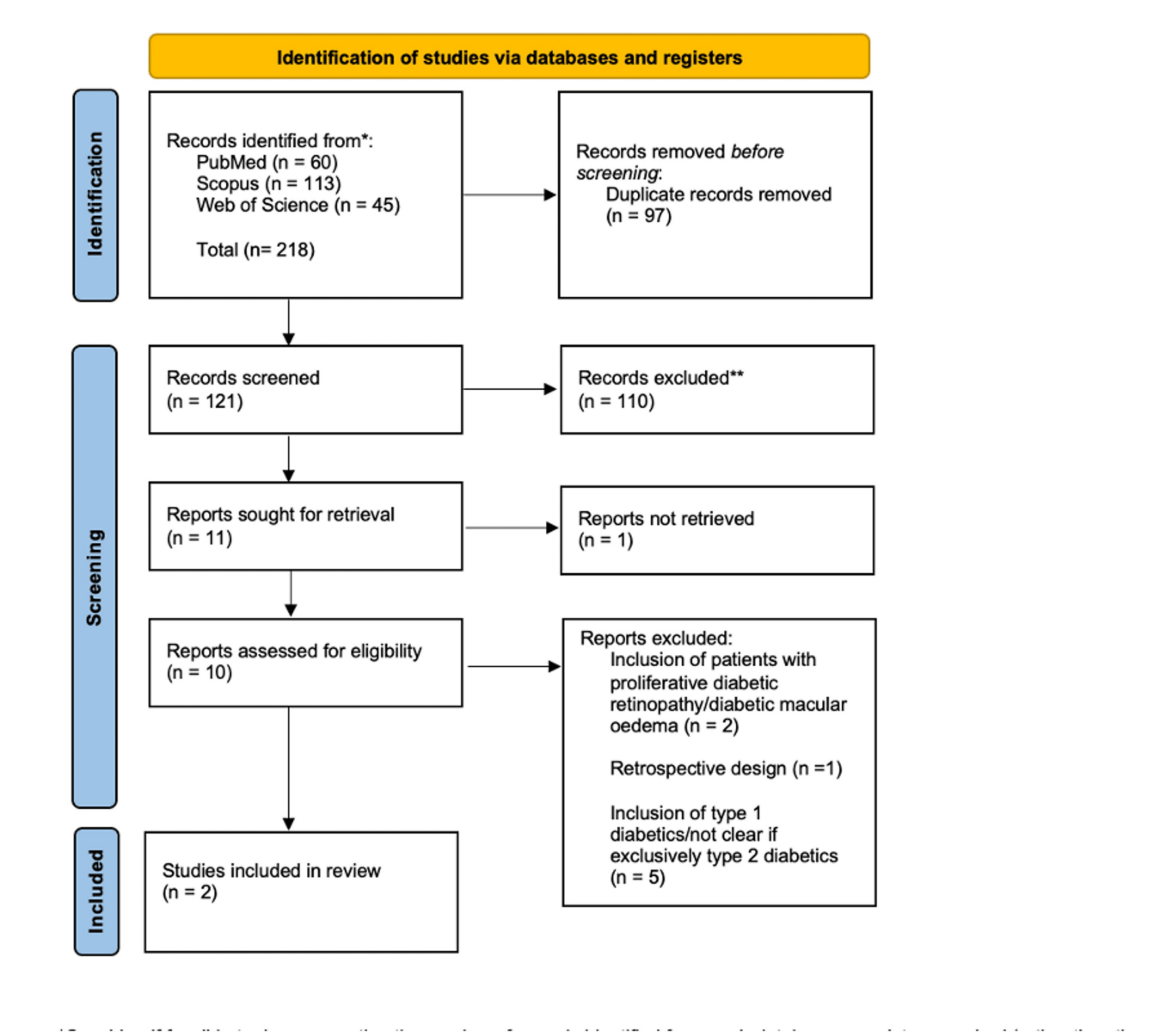
PRISMA diagram PRISMA: Preferred Reporting Items for Systematic Reviews and Meta-Analyses.

A total of 121 records were identified through searches of Web of Science, Scopus, and PubMed. After duplicate removal and title and abstract screening, 11 studies were retrieved for full-text assessment. Of these, eight were excluded for reasons including: retrospective study design, inclusion of type 1 diabetics, presence of diabetic retinopathy, use of combined surgical procedures, absence of a non-diabetic comparator, or lack of visual acuity outcomes relevant to the review question. Two studies met all eligibility criteria and were included in the qualitative synthesis. This is all illustrated in Figure 1.

Study Characteristics

The two included studies were published in 2022 and were conducted in India. Both were prospective comparative cohort studies evaluating postoperative outcomes following phacoemulsification with posterior chamber intraocular lens implantation. Across studies, a total of 137 eyes in adults with type 2 diabetes mellitus and 137 eyes in non-diabetic controls were analysed. Sample sizes were 74 and 200 eyes, and follow-up ranged from six weeks to three months. Surgical technique was standardised within each study and performed by a single surgeon.

Visual acuity outcomes were reported as best-corrected visual acuity (BCVA), in Snellen or converted logMAR notation, at early and short-term postoperative intervals. All studies explicitly excluded ocular comorbidities likely to affect visual prognosis, including other macular pathology, prior intraocular surgery, and significant intraoperative complications. Two studies additionally incorporated corneal endothelial cell analysis or macular thickness evaluation using optical coherence tomography. The characteristics and results of the studies are summarised in Table 2.

**Table 2:** Study characteristics and results DM: Diabetes mellitus, OCT: Optical coherence tomography, BCVA: Best-corrected visual acuity, CME: Cystoid macular edema.

Study	Country	Design	Sample size (DM/non-DM)	Follow-up timepoints	Visual acuity outcomes (as reported)	OCT/corneal outcomes (as reported)	Post-operative complications (as reported)
Mehra et al. 2022 [13]	India	Prospective comparative	37/37 patients (one eye each)	one week, six weeks	BCVA (logMAR): baseline 0.62±0.21 vs 0.71±0.18; one week 0.12±0.10 vs 0.12±0.10; six weeks 0.04±0.10 vs 0.04±0.09; intergroup p=0.423 at six weeks	Central retinal thickness increased in the diabetic group at six weeks (within-group p=0.047); between-group difference NS; no endothelial metrics	Clinical CME 1/37 in each group
Chaurasia et al. 2022 [14]	India	Prospective observational comparative	100/100 patients (one eye each)	one week, four weeks, three months	BCVA (logMAR): baseline 0.69 vs 0.67; one week 0.18 vs 0.15 (p=0.034); four weeks 0.02 vs 0.005 (p=0.001); three months 0.014 vs 0.003 (p=0.005)	Greater endothelial cell loss and greater central corneal thickness early in the diabetic group	Not reported

Risk of Bias

The studies were assessed using the Newcastle-Ottawa Scale. Both studies were judged to be at moderate risk of bias. Study limitations were primarily related to potential confounding inherent in non-randomised designs, limited control for systemic diabetic factors such as diabetes duration and glycaemic status, and short follow-up durations that may not reflect longer-term visual outcomes. The moderate risk of bias suggests that the findings should be interpreted with caution. While the included studies provide useful preliminary insights, the certainty of the evidence remains limited, and the results cannot be taken as definitive without larger, well-controlled prospective studies. A detailed summary of the risk-of-bias assessments is provided in Table 3.

**Table 3:** Risk of bias assessment

Study	Selection (4★ max)	Comparability (2★ max)	Outcome (3★ max)	Total Score (9★ max)
Mehra et al. 2022 [13]	4	1	1	6
Chaurasia et al. 2022 [14]	4	1	2	7

*Main findings*

Across the two included prospective comparative studies (a total of 274 eyes), postoperative visual acuity improved substantially after uncomplicated phacoemulsification in both people with type 2 diabetes without retinopathy and non-diabetic controls, with only small between-group differences over the first one to three months. Both studies restricted inclusion to patients with senile cataract, excluded any pre-existing retinal pathology (including diabetic retinopathy), and used a single experienced surgeon, thereby minimising baseline ocular confounders and surgical variability.

Baseline Characteristics

Baseline visual acuity was broadly comparable between diabetic and non-diabetic eyes. In Mehra et al. [13], diabetics actually presented with slightly better mean pre-operative BCVA than non-diabetics (0.62 ± 0.21 vs 0.71 ± 0.18 logMAR; p=0.050), whereas in Chaurasia et al., the pre-operative BCVA was almost identical in both groups (0.69 ± 0.21 vs 0.67 ± 0.20 logMAR; p=0.519). Taken together, these data suggest that at the time of surgery, visual disability from cataract was similar in diabetics without retinopathy and non-diabetics in the included cohorts.

Visual Acuity Outcomes

Both studies demonstrated marked and statistically significant improvement in BCVA after surgery within each group, but the pattern of between-group differences varied slightly. Mehra et al. [13] reported identical early postoperative acuity in diabetics and controls: at one week, mean BCVA improved to 0.12 ± 0.10 logMAR in both groups, and by six weeks to 0.04 ± 0.10 vs 0.04 ± 0.09 logMAR (overall p for between-group comparison =0.423). In contrast, Chaurasia et al. [14] found that although both groups gained excellent vision, non-diabetic eyes achieved slightly better acuity at each follow-up: at one week, BCVA was 0.15 ± 0.10 vs 0.18 ± 0.09 logMAR (p=0.034), at four weeks, 0.005 ± 0.02 vs 0.02 ± 0.02 logMAR (p=0.001), and at three months, 0.003 ± 0.02 vs 0.014 ± 0.03 logMAR (p=0.005) for non-diabetic and diabetic eyes, respectively.

When viewed together, these findings indicate that phacoemulsification restores high-quality vision in both groups; one study suggests complete equivalence, while the other demonstrates a small but statistically significant early advantage for non-diabetics, with absolute differences on the order of 0.01-0.03 logMAR (i.e., less than a Snellen line).

*Retinal Changes*

Structural outcomes help to contextualise these functional results. Mehra et al. [13] used OCT to evaluate central retinal thickness (CRT) and the incidence of cystoid macular oedema (CMO). CRT increased significantly from baseline to six weeks in diabetic eyes (212.7 ± 14.8 to 229.8 ± 50.3 µm; p=0.047), whereas the change in non-diabetic eyes was not statistically significant; however, mean CRT did not differ between groups at any time point (all p≥0.70). Clinical CMO occurred in 1/37 eyes (2.7%) in each group, so diabetes without retinopathy did not confer an excess CMO risk in this cohort. At six weeks, higher CRT correlated strongly with worse BCVA in both diabetics and non-diabetics, suggesting that macular thickening, when present, has similar visual consequences irrespective of diabetic status.

Corneal Changes

Chaurasia et al. [14] focused on corneal endothelial integrity and central corneal thickness (CCT) as potential determinants of visual recovery. Despite comparable pre-operative endothelial cell density and CCT, diabetic eyes experienced greater endothelial cell loss, higher coefficient of variation, lower hexagonality, and thicker corneas than non-diabetic eyes at one week and four weeks postoperatively, with most differences diminishing by three months. Over the same period, non-diabetic eyes consistently achieved marginally better BCVA than diabetic eyes, raising the possibility that subclinical corneal endothelial dysfunction in diabetes may contribute to slightly slower or less complete visual recovery, even in the absence of retinopathy.

Overall, integrating findings across both studies shows that in well-controlled type 2 diabetics without pre-existing retinal disease, phacoemulsification yields substantial and rapid gains in visual acuity that are broadly comparable to those achieved in non-diabetic patients. Where between-group differences are observed, they favour non-diabetics but are small in magnitude and occur in the context of more pronounced subclinical structural changes at both the macula and corneal endothelium in diabetic eyes.

Discussion

This systematic review evaluated postoperative visual acuity outcomes following phacoemulsification in patients with type 2 diabetes mellitus (T2DM) without clinical diabetic retinopathy or diabetic macular edema, compared with non-diabetic individuals. Across the two included prospective comparative cohort studies, totalling 137 eyes in diabetics and 137 in non-diabetics, the overall evidence supports that visually meaningful improvement is achieved in both groups. All studies demonstrated significant postoperative visual acuity gains, which aligns with current understanding of cataract surgery as an effective intervention even at early stages of diabetic ocular involvement.

The primary area of interest was whether diabetes without retinopathy confers measurable postoperative visual disadvantage. Findings were mixed: one study reported statistically significant but clinically small differences favouring non-diabetic patients at short-to-intermediate term follow-up, whereas one study reported no between-group difference at six weeks. Taken together, these data suggest that the presence of diabetes alone, in the absence of retinopathy or pre-existing maculopathy, does not meaningfully impair visual prognosis after phacoemulsification.

Secondary analytical outcomes offer further context. Diabetic patients exhibited higher rates of transient postoperative inflammation, greater early endothelial cell loss, and subclinical macular thickening, indicating a slightly heightened susceptibility to postoperative tissue stress. However, these anatomical changes seldom translated into functional deficits over the short follow-up period of available studies. The convergence of structural fragility with preserved visual function reinforces the need for vigilant monitoring but provides strong reassurance regarding patient-reported outcomes.

*Evidence Appraisal*

The internal validity of the included evidence warrants caution. Both studies were observational, with moderate risk of bias primarily due to potential confounding and limited follow-up duration. Glycaemic control, duration of diabetes, and cataract density were not consistently adjusted for in statistical analyses, despite a biological rationale for influence on healing [15]. The maximum follow-up of three months does not address longer-term outcomes such as posterior capsular opacification or development of postoperative diabetic macular oedema [16,17]. Heterogeneity in outcome reporting also limits formal quantitative synthesis. Despite these methodological constraints, findings from these studies align with broader epidemiological and clinical insights: good visual outcomes can be anticipated in diabetics free from retinal complications, provided meticulous perioperative planning and systemic metabolic optimisation are pursued.

Implications for Practice

From a clinical perspective, the findings of this review support the recommendation of phacoemulsification for individuals with type 2 diabetes mellitus who do not show any signs of diabetic retinopathy or macular disease. These patients can be counselled that their expected visual outcomes are comparable to those of non-diabetic individuals, although a slightly slower early postoperative recovery and a marginally increased risk of transient corneal or macular changes should be anticipated. However, corneal endothelial cell loss remains an issue even in manual small incision cataract surgery, another surgical technique [18]. Such observations reinforce the importance of attentive early postoperative monitoring in this group. Rigorous pre-operative retinal imaging, using fundus examination with or without optical coherence tomography, remains an essential component of clinical evaluation to confirm eligibility and provide accurate prognostic counselling [19]. Optimising glycaemic control before and after surgery also remains prudent, given its potential influence on healing responses and subclinical tissue stress [20].

Implications for Research

Future research should prioritise well-designed prospective studies with longer-term follow-up to distinguish early transient differences from delayed clinically significant sequelae in diabetic eyes without retinopathy. Standardisation of postoperative reporting, particularly the consistent use of logMAR visual acuity and OCT-derived macular metrics, would improve data synthesis and strengthen evidence for clinical decision-making. Furthermore, stratifying outcomes by factors such as the duration of diabetes, HbA1c levels, and systemic microvascular disease burden may help refine prognostic accuracy and facilitate more personalised perioperative planning.

## Conclusions

Phacoemulsification delivers excellent visual outcomes in patients with type 2 diabetes mellitus who do not exhibit diabetic retinopathy or macular disease, with postoperative results largely comparable to those of non-diabetic individuals. Although subtle differences in early healing responses and transient structural changes can occur, these do not appear to compromise short-term functional vision. The evidence therefore supports confident surgical management of visually significant cataract in well-screened diabetic patients, accompanied by careful perioperative monitoring and continued attention to metabolic control. Further research with extended follow-up and standardised reporting is warranted to confirm long-term equivalence and optimise care strategies for this growing patient population.

## Appendices

Date of search: 24th May, 2025

**Table 4:** PubMed search

No.	Search query	Results
#1	"Diabetes Mellitus"[MeSH] OR diabetes[tiab] OR diabetic[tiab] OR T1DM[tiab] OR T2DM[tiab]	931,644
#2	"Cataract Extraction"[MeSH] OR "cataract surgery"[tiab] OR “cataract extraction”[tiab] OR phacoemulsification[tiab] OR "lens extraction"[tiab] OR "cataract removal"[tiab] OR "intraocular lens implantation"[tiab] OR "IOL implantation"[tiab]	53,321
#3	non-diabetic[tiab] OR non-diabetes[tiab] OR “without diabetes”[tiab] OR “non diabetic”[tiab] OR “non diabetes”[tiab]	38,841
#4	"Visual Acuity"[MeSH] OR "visual outcome*"[tiab] OR "visual improvement"[tiab] OR "vision improvement"[tiab] OR "visual acuity"[tiab] OR "visual prognosis"[tiab] OR BCVA[tiab] OR UCVA[tiab]	144,186
#5	#1 AND #2 AND #3 AND #4	60

**Table 5:** Scopus search

No.	Search query	Results
#1	TITLE-ABS-KEY (diabetes) OR TITLE-ABS-KEY (diabetic) OR TITLE-ABS-KEY (T1DM) OR TITLE-ABS-KEY (T2DM)	1,414,623
#2	TITLE-ABS-KEY ("cataract surgery") OR TITLE-ABS-KEY (“cataract extraction”) OR TITLE-ABS-KEY (phacoemulsification) OR TITLE-ABS-KEY ("lens extraction") OR TITLE-ABS-KEY ("cataract removal") OR TITLE-ABS-KEY ("intraocular lens implantation") OR TITLE-ABS-KEY ("IOL implantation")	75,290
#3	TITLE-ABS-KEY (non-diabetic) OR TITLE-ABS-KEY (non-diabetes) OR TITLE-ABS-KEY (“without diabetes”) OR TITLE-ABS-KEY (“non diabetic”) OR TITLE-ABS-KEY (“non diabetes”)	46,701
#4	TITLE-ABS-KEY ("visual outcome*") OR TITLE-ABS-KEY ("visual improvement") OR TITLE-ABS-KEY ("vision improvement") OR TITLE-ABS-KEY ("visual acuity") OR TITLE-ABS-KEY ("visual prognosis") OR TITLE-ABS-KEY (BCVA) OR TITLE-ABS-KEY (UCVA)	208,288
#5	#1 AND #2 AND #3 AND #4	113

**Table 6:** Web of Science seatch

No.	Search query	Results
#1	AB=diabetes OR AB=diabetic OR AB=T1DM OR AB=T2DM	743,478
#2	AB="cataract surgery" OR AB=“cataract extraction” OR AB=phacoemulsification OR AB="lens extraction" OR AB="cataract removal" OR AB="intraocular lens implantation" OR AB="IOL implantation"	28,574
#3	AB=non-diabetic OR AB=non-diabetes OR AB=“without diabetes” OR AB=“non diabetic” OR AB=“non diabetes”	37,070
#4	AB="visual outcome*" OR AB="visual improvement" OR AB="vision improvement" OR AB="visual acuity" OR AB="visual prognosis" OR AB=BCVA OR AB=UCVA	82,709
#5	#1 AND #2 AND #3 AND #4	45
